# Selective Embolization in the Treatment of Pelvic Congestion Syndrome: A Comprehensive Case Report and Review of the Literature

**DOI:** 10.7759/cureus.79264

**Published:** 2025-02-19

**Authors:** Miguel A Peraza-Arjona, Victor M Ayuso-Diaz, Alfonso Peraza-Fernandez, Angelica Moreno-Enriquez, Mayra A Guachun-Guachun

**Affiliations:** 1 Angiology, Vascular and Endovascular Surgery, Elvia Carrillo Puerto Regional Hospital, Yucatán, MEX; 2 Surgery, Elvia Carrillo Puerto Regional Hospital, Yucatán, MEX; 3 General Practice, Genomic-Metabolic Unit, Marista University of Mérida, Yucatán, MEX; 4 Clinical Recruitment, Medical Care and Research, Yucatán, MEX; 5 Genetics and Molecular Biochemistry, Genomic-Metabolic Unit, Marista University of Mérida, Yucatán, MEX; 6 Angiology, Vascular and Endovascular Surgery, Cuenca Municipal Hospital, Cuenca, ECU

**Keywords:** case report, clinical outcomes, embolism, minimally invasive procedure, ovarian reflux, pelvic congestion syndrome, vulvar varicosities

## Abstract

Pelvic congestion syndrome is characterised by chronic pelvic pain lasting more than six months and is often underdiagnosed. Advanced imaging modalities play a crucial role in guiding further diagnostic evaluations and determining the appropriate treatment strategy. In the case presented, a phlebography with 3D reconstruction revealed ovarian vein reflux accompanied by an increased diameter, which confirmed the diagnosis. We present a case study of a 29-year-old woman who developed vulvar varicosities during her first pregnancy, was diagnosed with pelvic congestion syndrome in the postpartum period, and was successfully treated with endovascular therapy, with a year of follow-up.

## Introduction

Pelvic congestion syndrome is a common cause of chronic pelvic pain in women of reproductive age and represents a significant diagnostic and therapeutic challenge. It is characterised by the presence of persistent pain for more than six months without evidence of inflammatory disease. This pain can vary in intensity and is often worse during the premenstrual period, at night, or after activities such as prolonged walking or standing. In addition to pain, patients may present with symptoms such as dyspareunia, dysmenorrhoea, vulvar varicose veins, bladder irritability, rectal discomfort, and postcoital pain, making diagnosis based on clinical symptoms alone difficult [1,2].

The pathophysiology includes ovarian and pelvic venous reflux secondary to venous valve insufficiency or anatomical obstructions such as left renal vein compression syndrome (nutcracker syndrome) or left iliac vein compression syndrome (May-Thurner syndrome). These conditions must be carefully excluded by advanced imaging studies before a definitive diagnosis can be made and the most appropriate treatment selected [2-3].

In this context, the use of tools such as phlebotomography with three-dimensional reconstruction, conventional phlebography, and contrast-enhanced MRI have proven to be crucial in identifying structural and functional abnormalities of the pelvic venous system. In addition, a multidisciplinary approach involving gynaecology, interventional radiology, and other specialties is essential to optimise both diagnostic and therapeutic management of this complex pathology [1-3].

The present case highlights the importance of these combined strategies in the management of a Mexican patient who presented with this syndrome in the context of her first pregnancy. The clinical course, the interventions performed, and the results obtained illustrate the impact of a comprehensive approach on the quality of life of patients with this condition.

## Case presentation

A 29-year-old female patient with no known past medical history or comorbidities presented during the third trimester of her first pregnancy with progressive onset of lower limb heaviness, fatigue, dyspareunia, and prominent vulvar varicose veins. An initial laboratory assessment - including a complete blood count and coagulation studies - confirmed the absence of significant systemic or haematological disorders (see Table 1).

**Table 1:** Initial laboratory parameters

Parameter	Result	Reference Range
Red Blood Cells (RBC)	4.53	4.00 – 4.80 ×10^6/µL
Haemoglobin	12.5	12.0 – 14.0 g/dL
Haematocrit	38.2	38.0 – 45.0 %
White Blood Cells (WBC)	4.07	4.00 – 10.00 ×10^3/µL
Segmented Neutrophils	61	45 – 70 %
Absolute Lymphocytes	1180	1500 – 4500 cells/µL
Platelets	244	150 – 450 ×10^3/µL
Prothrombin Time (PT)	12.3	11.1 s
Prothrombin Activity (%)	83	Normal values may vary
International Normalized Ratio (INR)	1.11	0.9 – 1.1
Activated Partial Thromboplastin Time (aPTT)	37.5	29 s

Following this initial assessment, the patient reported significant discomfort associated with her symptoms. The Oswestry Disability Index (ODI) documented a baseline score of 48%, indicating moderate disability. In addition, an assessment using the Edinburgh Postnatal Depression Scale (EPDS) resulted in a score of 16, reflecting significant emotional impairment. These factors, combined with difficulties with activities of daily living - such as walking, prolonged standing and household tasks - had a clear negative impact on her quality of life.

Given the progression of her symptoms and the potential risks, her gynaecologist decided to terminate her pregnancy by elective caesarean section. After delivery and during the lactation period, the patient continued to experience heaviness and dyspareunia despite initial conservative management measures such as phlebotonics and venous hygiene. In addition, she reported significant difficulties in caring for her newborn, which exacerbated her stress levels, as evidenced by a score of 7/10 on the Visual Stress Scale (VSS).

**Figure 1:**
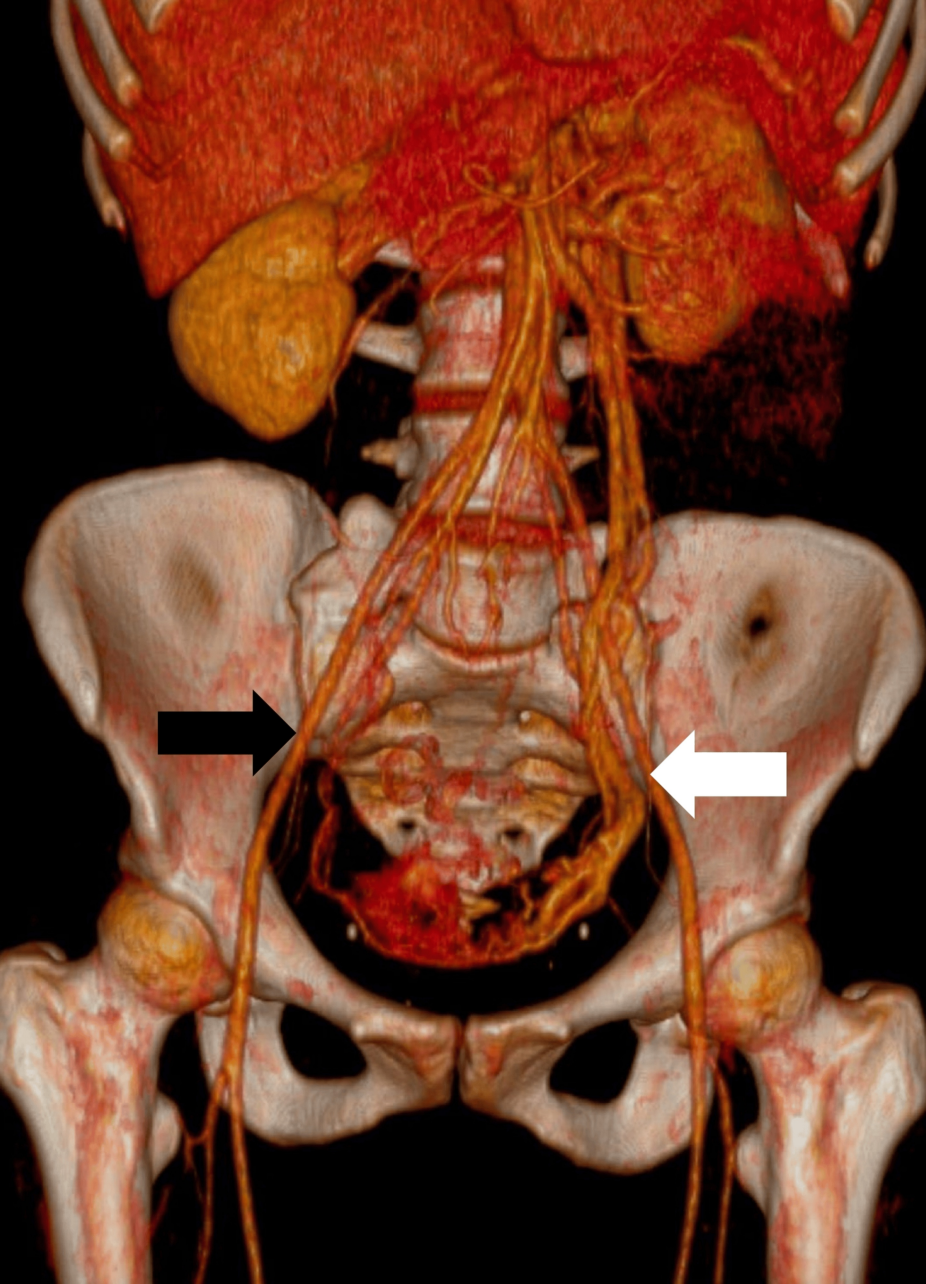
Phlebotomography with 3D reconstruction showing ovarian vein reflux with an increased diameter (black arrow). Phlebotomography with a three-dimensional reconstruction showing reflux in the left ovarian vein. There is a significant increase in venous diameter, indicated by the white arrow, which is characteristic of pelvic congestion syndrome. This finding supports the presence of pelvic venous insufficiency and its contribution to the patient's clinical symptoms.

As her symptoms did not resolve completely, a phlebotomography was performed, which revealed a marked reflux in the left ovarian vein (Figure 1), confirming the diagnosis of pelvic congestion syndrome.

**Figure 2:**
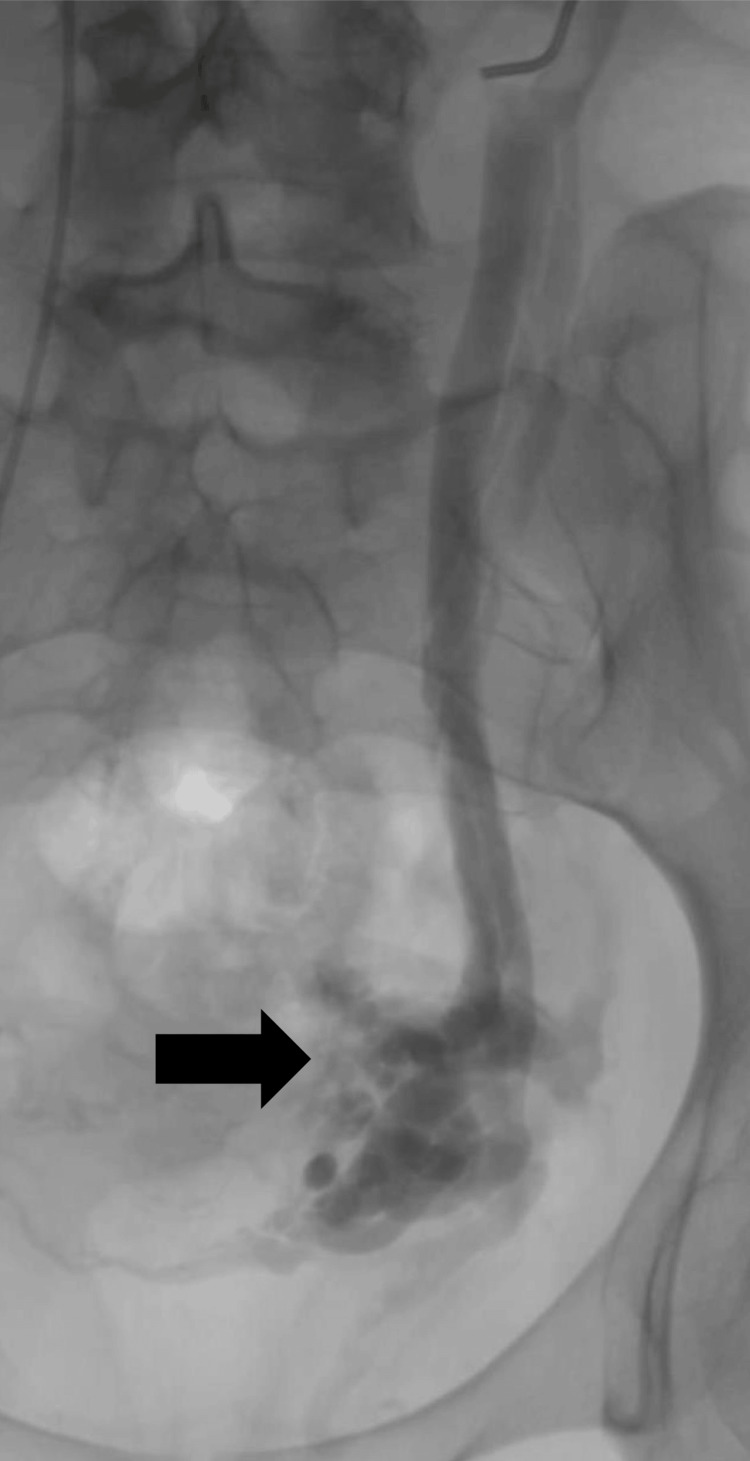
Initial phlebography shows the femoral vein approach, 12 mm left ovarian vein, and contrast reflux to the pelvic veins (black arrow) The initial phlebography illustrates the femoral vein approach and shows a dilated left ovarian vein with a diameter of 12 mm. Contrast reflux into the pelvic veins is seen, indicated by the black arrow, confirming the venous involvement characteristic of pelvic congestion syndrome.

Once the diagnosis was established, and after waiting six months to complete the lactation period, definitive treatment by endovascular embolization was chosen. The procedure was performed under local anaesthesia via the right femoral vein. Initial phlebography revealed significant dilatation of the left ovarian vein (Figure 2). Selective cannulation of the affected vein was achieved using a Cobra-type catheter. A catheter was then advanced over a hydrophilic guidewire and embolic agents were administered by placing five coils: two 6 mm x 20 cm, two 8 mm x 40 cm, and one 5 mm x 10 cm. 

**Figure 3:**
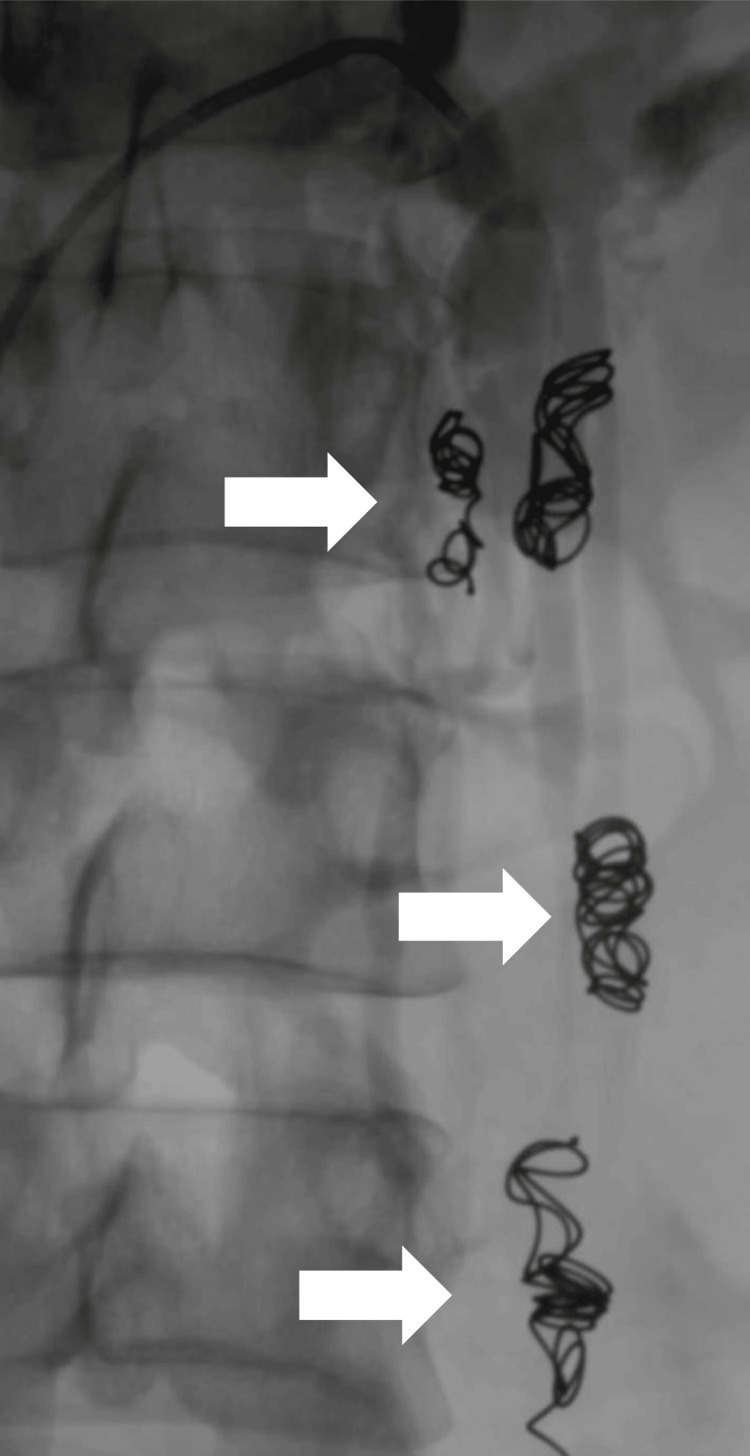
Post-embolization control phlebography with coils (white arrows: corroborating the absence of reflux and permeability of left renal vein) Post-embolisation control phlebography shows adequate placement of the coils (white arrows), confirming the absence of venous reflux in the pelvic region and preservation of patency of the left renal vein. These findings confirm the technical success of the endovascular approach.

The immediate post-procedure result was satisfactory, achieving complete closure of the left ovarian vein with preservation of patency of the left renal vein. A subsequent phlebography corroborated the correct placement of the coils and the absence of venous reflux in the pelvic region (Figure 3). During clinical follow-up, the patient experienced a marked improvement in symptomatology. Pelvic pain, previously assessed by visual analog scale (VAS) with a score of 8/10, decreased to 2/10 at one-month post-procedure and reached 0/10 at one year. The heaviness in the lower extremities subsided completely, allowing the patient to resume daily activities without functional limitations, such as caring for her child and performing household chores. 

**Figure 4:**
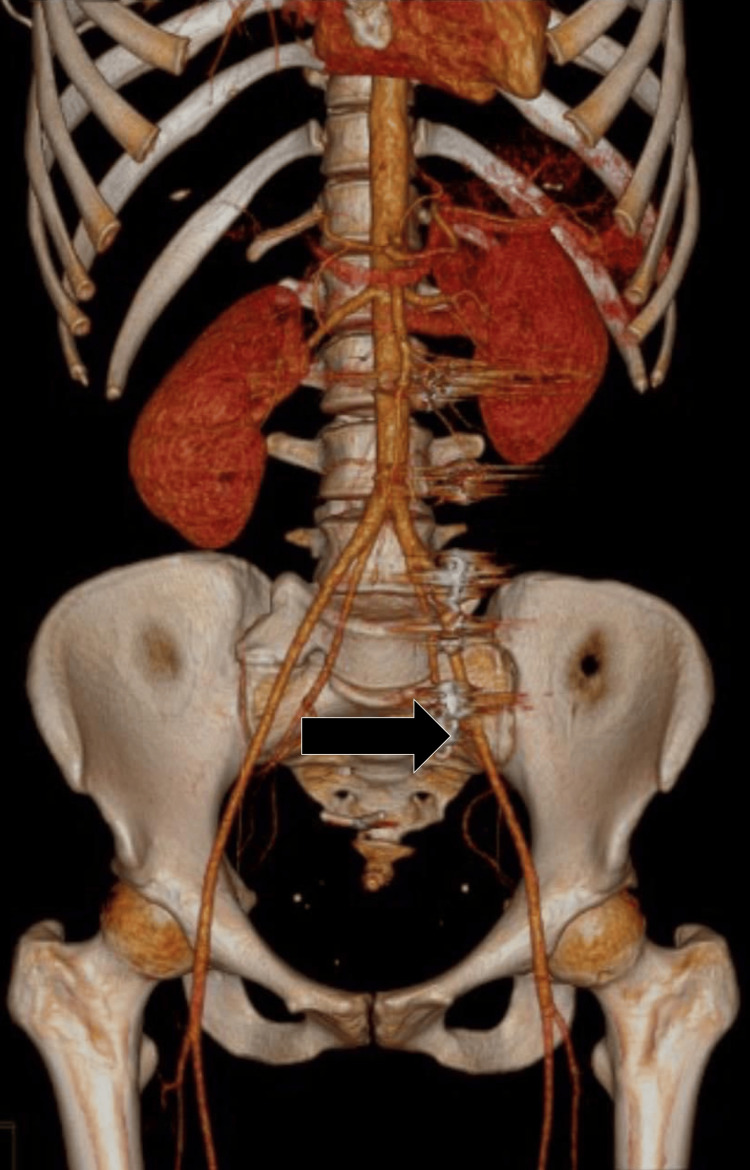
Control phlebotomography with 3D reconstruction (black arrow) Control phlebotomography with 3D reconstruction one year after embolization shows the absence of ovarian venous reflux. The vascular anatomy appears to be fully restored, with no signs of recurrence or collateral venous development, confirming the long-term efficacy of the treatment.

One year on from the procedure, phlebotomography with 3D reconstruction were performed to confirm complete resolution of venous reflux. There was no evidence of recurrence and adequate vascular anatomy was confirmed in the operated region (see Figure 4). Follow-up laboratory parameters remained stable, showing preserved renal function and metabolic balance. This is consistent with the resolution of pelvic congestion syndrome (Table 2).

**Table 2:** Follow-up renal and metabolic laboratory parameters The patient's renal and metabolic laboratory parameters are presented, demonstrating stable kidney function, glucose metabolism, and nitrogen balance one year after endovascular treatment. These findings support the resolution of pelvic congestion syndrome without secondary metabolic complications.

Parameter	Result	Reference Range
Serum Glucose (colourimetric, VITROS)	96 mg/dL	70–99 mg/dL (fasting)
Serum Creatinine (kinetic, VITROS)	0.57 mg/dL	0.5–1.1 mg/dL (female)
Estimated Glomerular Filtration Rate (eGFR) (CKD-EPI)	127 mL/min/1.73 m²	> 90 mL/min/1.73 m²
Blood Urea Nitrogen (BUN) (urease–colourimetric)	17.1 mg/dL	7–20 mg/dL
Urea (urease–colourimetric)	36.7 mg/dL	15–40 mg/dL
BUN/Creatinine Ratio (calculated)	30	10–20
Serum Uric Acid (colourimetric, VITROS)	4.3 mg/dL	2.6–6.0 mg/dL (female)

## Discussion

Pelvic congestion syndrome is a relatively common condition in women of childbearing age, affecting up to 30% to 40% of women with chronic pelvic pain without an alternative diagnosis. It is characterised by dilatation and dysfunction of the pelvic veins, associated with the presence of chronic pain, dyspareunia, vulvar varicosities, and symptoms related to the pelvic venous system [4,5]. The prevalence of this condition is higher in multiparous women, particularly those with a history of multiple pregnancies [6,7]. The increase in venous capacity during pregnancy may contribute to the development of chronic venous incompetence, which persists after pregnancy. Other risk factors include obesity, a family history of varicose veins, and the presence of polycystic ovary syndrome, which increase the likelihood of developing this condition [7,8].

Despite the high prevalence of this syndrome, it remains a diagnostic challenge. Symptoms such as chronic pelvic pain, lower limb heaviness, and dyspareunia can be confused with other gynaecological conditions such as endometriosis, uterine fibroids, or chronic pelvic infections [9]. In the case of the patient presented here, the situation was particularly complex. During her first pregnancy, she developed typical symptoms such as lower limb heaviness, dyspareunia, and the appearance of vulvar varicose veins in the third trimester. Because of this clinical presentation, it was decided to carry out a thorough evaluation to rule out malignant or infectious gynaecological conditions. After delivery, despite conservative measures such as phlebotonic drugs and venous hygiene recommendations, the symptoms persisted, leading to phlebography, which confirmed the presence of venous dilatation in the ovarian veins and venous reflux, thus confirming the diagnosis.

The diagnosis of this condition has been greatly improved by the use of advanced imaging techniques such as venography and phlebography. These allow detailed assessment of the affected veins, and the sensitivity of these techniques for detecting venous reflux is high. Phlebography has shown a sensitivity of 100% for the left ovarian vein and 67% for the right ovarian vein [9]. In this case, phlebography showed significant dilatation and reflux in the left ovarian vein, which allowed accurate diagnosis and planning of appropriate treatment. In addition, venography helped to rule out other possible causes of the symptoms, such as endometriosis or fibroids, which facilitated an accurate therapeutic approach.

In terms of treatment, transcatheter embolisation of the ovarian veins has been established as a first-line option for the management of this condition in women with severe symptoms, especially when conservative treatments have not been effective. This procedure has several advantages over other therapeutic options, such as surgical ligation of the ovarian veins, in that it is less invasive, has a lower complication rate, and faster recovery. Embolization has been shown to have a success rate of over 85% in relieving symptoms, and a low recurrence rate has been reported, making it the preferred treatment option in many specialist centres [10,11].

In this patient, a Cobra 5-F catheter was used to select the left renal vein, allowing precise embolization of the left ovarian vein by placement of 6 mm x 20 cm, 8 mm x 40 cm, and 5 mm x 10 cm coils, resulting in successful occlusion of the affected vein. The choice of these coils is based on their proven efficacy and ability to conform to the individual venous anatomy of each patient. The use of these high-quality devices is essential to minimise complications such as coil migration or local thrombophlebitis which, although rare, can occur in cases with complex venous anatomy [12]. In this patient, embolization resulted in almost immediate resolution of symptoms, with significant improvement in pelvic pain and dyspareunia, confirming the efficacy of this treatment.

Importantly, transcatheter embolisation not only relieves symptoms but also improves patients' quality of life by resolving persistent problems such as vulvar varicose veins and lower limb heaviness. The results obtained in this case are in line with the current literature, which reports success rates of more than 90% in reducing symptoms, with a low risk of complications and a recurrence rate that is generally less than 10%. The combination of advanced diagnostic techniques and the use of high-quality embolic material proved to be critical to the successful treatment of this patient [13].

## Conclusions

Transcatheter embolisation of the ovarian veins is the most effective and least invasive therapeutic option for treating pelvic congestion syndrome. It significantly relieves symptoms and improves patients' quality of life. This treatment is highly effective and provides a long-term solution with low recurrence rates.Thorough assessment and diagnostic confirmation using advanced imaging techniques, together with the use of high-quality medical materials, are key factors in successful treatment. This approach should be considered the treatment of choice for patients with persistent or severe symptoms. Research and development of minimally invasive techniques for the management of this complex disease must continue.
